# Effects of Basal Defoliation on Wine Aromas: A Meta-Analysis

**DOI:** 10.3390/molecules23040779

**Published:** 2018-03-28

**Authors:** Yu Wang, Lei He, Qiuhong Pan, Changqing Duan, Jun Wang

**Affiliations:** 1 Center for Viticulture & Enology, College of Food Science and Nutritional Engineering, China Agricultural University, Beijing 100083, China; wangyu_0919@cau.edu.cn (Y.W.); helei@cau.edu.cn (L.H.); panqh@cau.edu.cn (Q.P.); duanchq@vip.sina.com (C.D.); 2 Key Laboratory of Viticulture and Enology, Ministry of Agriculture, Beijing 100083, China

**Keywords:** basal defoliation, wine aroma, meta-analysis

## Abstract

Basal defoliation, as one of the most common viticulture management practices to modify fruit zone microclimates, has been widely applied aiming at improving wine quality. Wine aroma contributes greatly to wine quality, yet the effects of basal defoliation on wine aromas show discrepancies according to previous studies. This study is a meta-analysis performed to dissect the factors related to the influence of basal defoliation on volatile compounds in wine. Timing of basal defoliation plays an important role in the concentration of varietal aromas in wine. Pre-veraison defoliation induces an increase in *β*-damascenone and linalool as well as a reduction in 3-isobutyl-2-methoxypyrazine (IBMP). The effects of basal defoliation on certain volatile compounds relative to fermentation aromas in wine (1-hexanol, *β*-phenylethanol, 2-phenylethyl acetate, decanoic acid, and ethyl octanoate) depend on grape maturity. There are also other factors, such as cultivar and climate conditions, that might be responsible for the effect of basal defoliation on wine aromas. The concentrations of isobutanol, isoamyl alcohol, hexanoic acid, and octanoic acid as well as ethyl isobutyrate, ethyl hexanoate, ethyl isovalerate, and ethyl decanoate in wine are not markedly affected by basal defoliation. Due to limited studies included in this meta-analysis, more trials are needed to confirm the current findings.

## 1. Introduction

Basal defoliation, defined as the practice of leaf removal in the fruit zone, is one of the most common viticulture management practices to modify fruit zone microclimates (sunlight exposure, fruit zone temperature, and fruit zone air circulation). Previous studies have demonstrated that basal defoliation was effective against reducing foliage over, enhancing cluster light exposure and canopy porosity, and controlling the incidence of disease [1,2,3]. Changes in fruit zone microclimates are believed to impact quality-associated primary and secondary metabolites in grape and wine composition [4,5,6]. Sunlight-exposed clusters by basal defoliation are generally characterized by higher sugars, anthocyanins, flavonols, and lower malic acid and titratable acidity compared with shaded clusters [7,8]. Therefore, in cool regions where low heat accumulation, high humidity, and rainfall are common, basal defoliation is widely used to advance berry ripening [9]. Nevertheless, under the background of global warming, the rise in average temperature and the associated increase in heat accumulation have caused earlier harvest dates in many regions [10]. Recently, late apical defoliation, removing leaves located above the bunch zone between veraison and harvest, has been reported as an effective method to delay harvest. Since young leaves at the top of the main shoot are the main contributors to the accumulation of sugars in the fruit between veraison and harvest, this technique could lead to source limitation and cause a delay of grape ripening [11,12,13]. Traditional basal defoliation, in warm regions, applied at veraison is more likely to lead to berry sunburn, which is unfavorable for the biosynthesis of anthocyanins [14,15]. Given this, an innovative viticultural technique of basal defoliation applied before flowering has been developed. This technique can not only influence berry composition by altering source-to-sink ratio, cluster compactness, and fruit exposure, but also prevent bunches from being subjected to over-exposure along with high temperatures in the hottest part of the season when lateral leaves grow back and partially shade bunches [16]. Additionally, several studies have documented that both the timing and severity of basal defoliation has an important effect on berry composition [17,18,19]. Therefore, appropriate timing and the extent of basal defoliation should be investigated based on the specific climate conditions.

Improvement of wine bouquet is of great interest to viticulturists and winemakers due to their importance to wine quality. Generally, wine aroma can be categorized as varietal aromas (terpenes, norisoprenoids, and methoxypyrazines), fermentation aromas (higher alcohols and their acetates as well as fatty acids and their ethyl esters) and aging aromas (volatile phenols). Varietal aromas of wine mainly derive from grapes and are subjected to genotypic and environmental factors (light, temperature, and water availability) [20]. Given this, basal defoliation can be an effective practice on directly modifying wine varietal aromas. Fermentation aromas are formed via fatty acid metabolism or amino acid metabolism by yeast activity during fermentation [21]. Furthermore, several studies have demonstrated that fatty acids and amino acids are sensitive to environmental factors. Fatty acids in berries have shown diverse behaviors in different training systems [22], for example, and concentrations of amino acids in berries have been altered by sunlight exposure [23,24]. Thus, it is possible that fermentation aromas can be affected by basal defoliation by altering their substrate levels. Many studies have recently been conducted to investigate the influence of basal defoliation on volatile compounds in grape and wine, but discrepancies exist among these studies. The controversial results across these studies indicate that the grape cultivar or clone [25,26,27], the climate condition [28], grape maturity [29], and the timing and severity of defoliation [28,30,31] might be responsible for the varied effects of basal defoliation on the aromatic properties of grape and wine.

Meta-analysis, using statistical methods to combine data from multiple experiments, is an objective and quantitative approach for summarizing results of published studies on the same topic. The authors of the current study collected and analyzed the data from previous studies investigating the effects of basal defoliation on wine aromas, with the aim to dissect the factors relative to these effects. Both the aromas originating from the grape and the aromas mainly produced during fermentation and processing are discussed. The findings of the present study are intended to provide a theoretical basis for viticulturists and winemakers to manipulate wine volatile profiles by appropriate defoliation.

## 2. Results and Discussion

### 2.1. The Effects of Basal Defoliation on Varietal Aromas

#### 2.1.1. C_13_-Norisoprenoids

C_13_-norisoprenoids have been identified as important volatile compounds in wine due to their low olfactory thresholds and pleasant smell. Though relatively lower levels of free C_13_-norisoprenoids are detectable in juice, the majority of C_13_-norisoprenoids in wine are derived from non-volatile C_13_-norisoprenoid glycosides, which can be released during the process of winemaking or storage [32]. Carotenoids serving as the precursors to both free and bound C_13_-norisoprenoids can be catalyzed by carotenoid cleavage dioxygenase (CCD) to produce aroma apocarotenoids including *β*-damascenone, *β*-ionone, geranylacetone, *α*-ionone, vitispirane A, vitispirane B, and TDN [33,34]. Attention was paid to two C_13_-norisoprenoids, *β*-damascenone and *β*-ionone. *β*-Damascenone contributes to floral, sweet, and fruity notes of wine, and it can enhance fruity notes of ethyl cinnamate and caproate as well as mask the herbaceous aroma of 3-isobutyl-2-methoxypyrazine (IBMP) in wine [35]. *β*-Ionone has also been widely studied for its typical violet odor [36,37].

**Figure 1:**
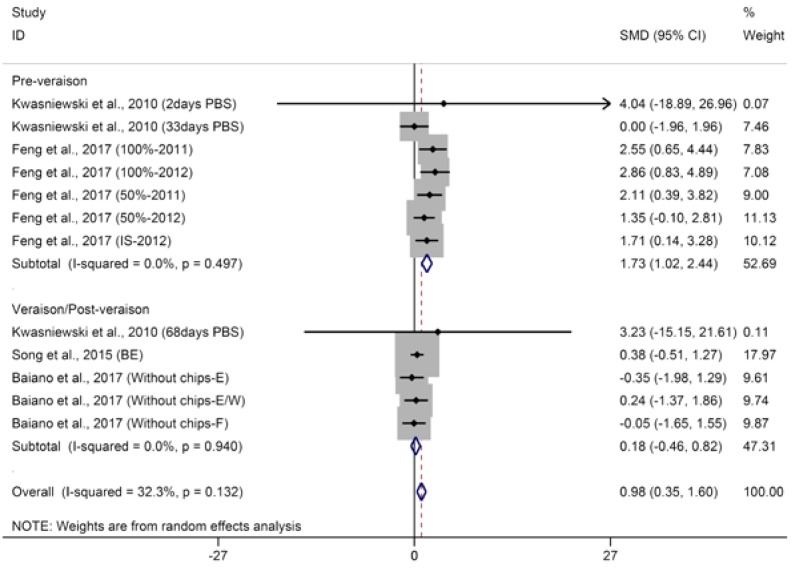
Meta-analysis for the effects of basal defoliation on *β*-damascenone in wine, of which trials were categorized based on the timing of basal defoliation (pre-veraison and veraison/post-veraison).

The aggregated results of meta-analysis for *β*-damascenone suggest that basal defoliation significantly improves the concentration of *β*-damascenone in wine (SMD = 0.81, 95% CI = 0.17–1.45, *p* = 0.01, *p* for heterogeneity = 0.05, *I*^2^ = 43%). Subgroup analyses were further conducted based on the timing of basal defoliation applied: pre-veraison (early) and veraison/post-veraison (late). Early defoliation significantly increases the concentration of *β*-damascenone in wine (SMD = 1.34, 95% CI = 0.35–2.32, *p* = 0.008, *p* for heterogeneity = 0.03, *I*^2^ = 55%). These noteworthy increases have only been reported by Feng et al. [28], who observed that industry-standard (IS) defoliation treatment is a major contributor to heterogeneity. Following removal of the heterogeneous observations, positive effects on *β*-damascenone in wine caused by early defoliation were not heterogeneous (Figure 1, SMD = 1.73, 95% CI = 1.02–2.44, *p* < 0.001, *p* for heterogeneity = 0.50, *I*^2^ = 0%). Several studies have confirmed that early defoliation can enhance the level of *β*-damascenone in wine [26,29]. Furthermore, higher defoliation levels cause greater concentrations of *β*-damascenone. The increase in *β*-damascenone levels induced by early defoliation is closely related to the fact that early defoliation significantly increases specific carotenoids in grapes [4,38]. Carotenoids are believed to be the essential components of all photosynthetic organisms, which participate in light harvesting and photoprotection [39]. A major regulatory mechanism of carotenoid genes appears to be developmentally regulated, and environmental factors such as light exposure also influence the expression of related genes [4]. Distinct increases of *β*-damascenone induced by pre-veraison defoliation might derive from higher substrate availability, since pre-veraison berries are photosynthetically active [40]. Contrarily, Kwasniewski et al. [38] reported that early defoliation has no significant effect on the concentration of *β*-damascenone in wine. Moreover, Verzera et al. [29] proposed that the eliminated effect of early defoliation on *β*-damascenone in the wine of second harvest that they observed (°Brix of grape approximately 24.6) was probably due to the excessive sun exposure of the clusters. A hidden effect of early defoliation on *β*-damascenone might also be attributed to the interference of cultivar genotype or climate factors of the experimental region [18,28]. Regarding the late defoliation subgroup, no differences were found among control and defoliated wines in concentrations of *β*-damascenone (Figure 1, SMD = 0.18, 95% CI = −0.47–0.82, *p* = 0.59, *p* for heterogeneity = 0.88, *I*^2^ = 0%). Regarding *β*-ionone in wine, meta-analysis indicates that basal defoliation significantly increases its concentration (Figure S1, SMD = 0.69, 95% CI = 0.22–1.15, *p* = 0.004, *p* for heterogeneity = 0.82, *I*^2^ = 0%); however, this result needs to be rigorously tested due to the limited number of studies.

#### 2.1.2. Terpenes

Terpenes are considered to be characteristic aroma contributors to the Muscat varieties, and play an important role in non-Muscat grapes and wines [41,42]. The most predominant terpenes in grape and wine are linalool, geraniol, limonene, *α*-terpineol, and nerol, which occur at low concentrations but have important effects on the sensory characteristics of wine. Therefore, winemakers are interested greatly in increasing the terpenes level in grapes and wines by various viticulture and wine-making techniques. The differences between the concentrations of these compounds can also be used to distinguish different varieties [43]. The compounds of terpenes such as linalool and geraniol have received more attention due to their sensory contribution.

**Figure 2:**
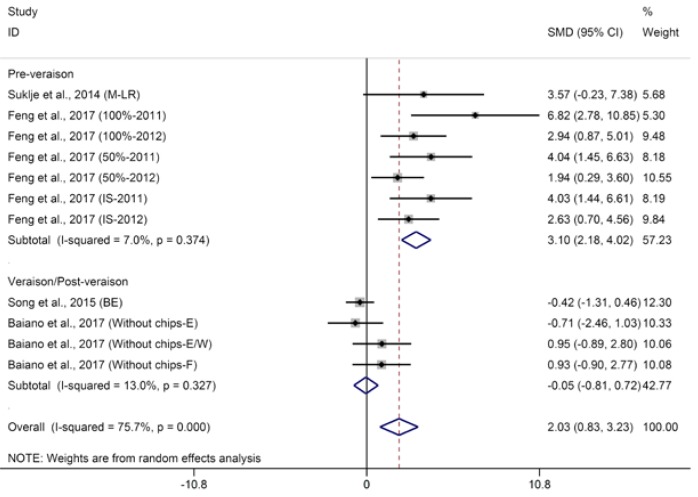
Meta-analysis for the effects of basal defoliation on linalool in wine, of which trials were categorized based on the timing of basal defoliation (pre-veraison and veraison/post-veraison).

The aggregated results of meta-analysis for linalool in wine suggest that defoliation significantly improves the concentration of linalool in wine with moderate heterogeneity (SMD = 1.53, 95% CI = −0.23–7.38, *p* = 0.002, *p* for heterogeneity < 0.001, *I*^2^ = 76%). Subgroup analyses were conducted based on the timing of basal defoliation applied. Regarding the pre-veraison subgroup, defoliation significantly increases the linalool level (*p* < 0.001, *p* for heterogeneity < 0.001, *I*^2^ = 76%). Sensitivity analysis indicates that the observations reported by Vilanova et al. [30] contribute to a greater proportion of heterogeneity in the subgroup of pre-veraison. After the study of Vilanova et al. [30] is removed, meta-analysis shows that early defoliation significantly increases linalool with low heterogeneity (Figure 2, SMD = 3.10, 95% CI = 2.18–4.02, *p* < 0.001, *p* for heterogeneity = 0.37, *I*^2^ = 7%). The authors’ meta-analysis results are in accordance with a recent study that demonstrated that sunlight exposure is essential to the biosynthesis and accumulation of linalool in berries [44]. Additional to the studies included in the meta-analysis, other studies found that the effect of early defoliation on linalool levels in wine depends on grape cultivar and the period of grape harvest. To be specific, Hernandez-Orte et al. [26] found that early-defoliation-treated wines Gewuztraminer and Chardonnay have higher amounts of linalool than did the control wines, while the opposite results were found in Merlot and Tempranillo wines. In Verzera et al.’s study [29], early defoliation significantly increased the concentration of linalool in wines of first harvest (°Brix of approximately 24.8), but it had no effect on linalool levels in the wine of second harvest (°Brix of grape approximately 24.6). Regarding the late defoliation subgroup, no marked differences were found among treatment and control wines for linalool concentrations (Figure 2, SMD = −0.05, 95% CI = −0.81–0.72, *p* = 0.90, *p* for heterogeneity = 0.33, *I*^2^ = 13%). The meta-analysis for geraniol indicates that basal defoliation leads to higher geraniol in wine with moderate heterogeneity (Figure S2, SMD = 1.10, 95% CI = 0.33–1.86, *p* = 0.005, *p* for heterogeneity = 0.02, *I*^2^ = 56%). Song et al. [45] reported that basal defoliation led to higher geraniol levels compared to controls and proposed that higher geraniol levels in wines can be attributed to higher grape maturity (°Brix of treated grapes approximately 23.6 versus that of controls being approximately about 21.7) as a result of sun exposure treatment [46]. However, higher concentrations of geraniol were also observed by Feng et al. [28] in 2011 in defoliated wines compared with control wines, even though basal defoliation had no evident effect on grape maturity. Additionally, Feng et al. [28] found that basal defoliation had no significant effect on geraniol levels in 2012 wines, which agreed with the study of Baiano et al. [31]. The two seasons exhibited distinct weather conditions in the study of Feng et al. [28]*:* the 2012 season was characterized by a warmer and drier climate than the 2011 season, and the weather variations might have been responsible for the varied effect of basal defoliation on geraniol levels in the wines of the two seasons [28]. Additionally, the influence of basal defoliation on geraniol might be altered by cultivar factors [26]. Overall, terpenes are present mainly in glycoside precursor form in grapes or juice and can be released by enzymatic and acidic hydrolysis to the corresponding free form during wine making [47]. The increase in principal glycoside terpene precursors induced by pre-veraison defoliation might lead to a higher amount of linalool and geraniol in wine [1]. However, due to the limited numbers of studies included in this meta-analysis, more precise and comprehensive experiments should be carried out to verify the previous results and explain the heterogeneity caused by the grape cultivar, the harvest date, and the timing of basal defoliation applied to finally benefit the wine industry.

#### 2.1.3. Methoxypyrazines

**Figure 3:**
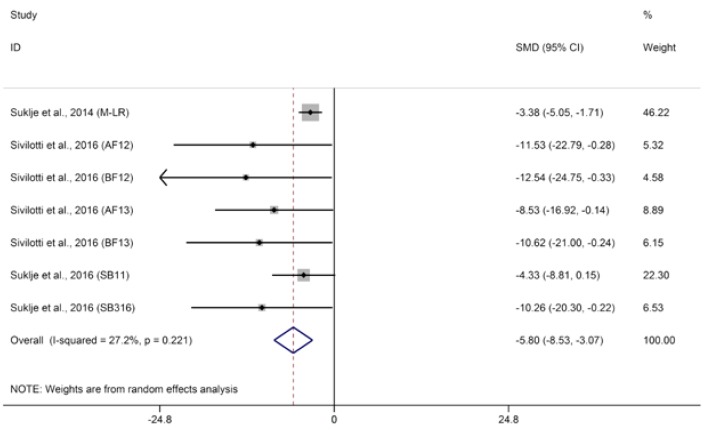
Meta-analysis for the effects of basal defoliation on 3-isobutyl-2-methoxypyrazine (IBMP) in wine.

The odor of methoxypyrazines is generally described as vegetal and bell pepper-like [48]. Although persisting at low concentrations, methoxypyrazines play an important role in the flavor of wine due to its quite low odor threshold in wine (~1 ng/L) [49]. Excessive methoxypyrazines in wine can mask floral and fruity notes of wine, which leads to unpleasant vegetative sensory notes especially in red wine [50]. Methoxypyrazines in wine are extracted from grape skins and stems during fermentation [51]. Among methoxypyrazines, 3-isobutyl-2-methoxypyrazine (IBMP) is most often reported above its threshold in wine. Koch et al. [48] observed that berry IBMP accumulated subsequent to berry set, peaked prior to the onset of ripening, and subsequently decreased until harvest. Increasing light intensity before ripening inhibited IBMP accumulation in berries, while light modulation during ripening did not significantly affect IBMP concentrations in the berries at harvest. Several studies also confirmed that basal defoliation applied before veraison significantly reduces the concentration of IBMP in berries at harvest [5,52,53]. Thus, it was clear that early basal defoliation leads to lower concentrations of IBMP in wines compared with the control treatment (Figure 3, SMD = −5.80, 95% CI = −8.53–3.07, *p* < 0.0001, *p* for heterogeneity = 0.22, *I*^2^ = 27%). However, due to the scarcity of papers investigating the effect of basal defoliation on IBMP in wine, more studies should be conducted to verify these meta-analysis results.

### 2.2. Effects of Basal Defoliation on Aromas Related to Fermentation

#### 2.2.1. C_6_-Compounds

**Figure 4:**
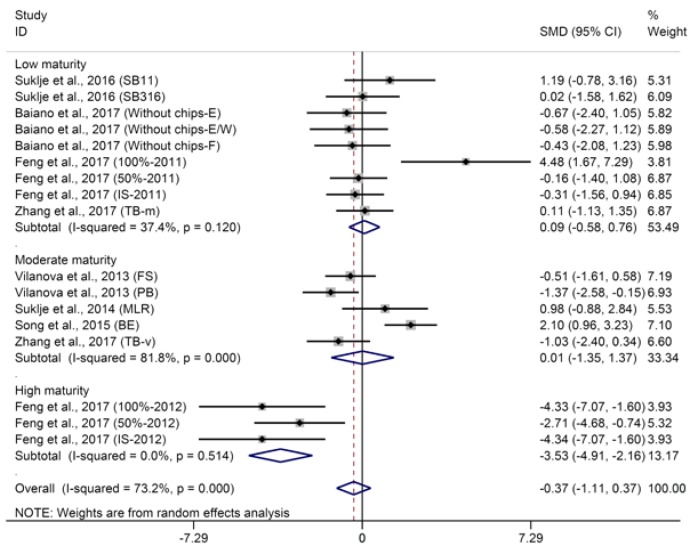
Meta-analysis for the effects of basal defoliation on 1-hexanol in wine, of which trials were categorized based on the berry maturity level (low maturity of °Brix < 23, moderate maturity of 23 < °Brix < 25, high maturity of °Brix > 25).

Six carbon (C_6_) compounds are referred to as “green leaf volatiles” (GLVs) since these compounds are characterized as “herbaceous” and “green” aromas. C_6_ compounds mainly consist of alcohols, aldehydes, and esters, which are generated through an oxylipin pathway in response to mechanical damage, fungal or bacterial infection, and abiotic stress such as high light, temperature, and drought [54]. Typical C_6_ alcohols mainly include 1-hexanol, (*E*)-3-hexenol, and (*Z*)-3-hexenol. Among these compounds, 1-hexanol is prevalent in wines and arises from the 1-hexanol present in grapes and from the transformation of hexanal, (*E*)-2-hexenol, and (*Z*)-2-hexenol during fermentation [55]. The aggregated results of the meta-analysis shows that basal defoliation has no significant influence on the concentration of 1-hexanol in wine (SMD = −0.37, 95% CI = −1.11–0.37, *p* = 0.33), whereas the pooled results were heterogeneous (*p* for heterogeneity < 0.001, *I*^2^ = 73%). Meta-regression analysis suggests that berry maturity level (shown in Section 3.2) is the main source of heterogeneity (*p* = 0.015). Therefore, further subgroup analysis was conducted to explore the effects of basal defoliation on 1-hexanol in wines from grapes with different maturities. Basal defoliation has no significant effect on 1-hexanol in wines from grapes with low maturity (Figure 4, SMD = 0.09, 95% CI = −0.58–0.76, *p* = 0.79, *p* for heterogeneity = 0.12, *I*^2^ = 37.4%), while it significantly decreases the concentration of 1-hexanol in wines from grapes with high maturity (Figure 4, SMD = −3.43, 95% CI = −4.91–2.16, *p* < 0.001, *p* for heterogeneity = 0.51, *I*^2^ = 0%). Verzera et al. [29] also found that the effect of basal defoliation on 1-hexanol in wine depends on the period of the grape harvest. Regarding the wines from grapes with moderate maturity, basal defoliation produces varied effects on 1-hexanol (Figure 4, SMD = 0.01, 95% CI = −1.35–1.37, *p* = 0.99, *p* for heterogeneity < 0.001, *I*^2^ = 82%). Vilanova et al. [30] proposed that the significant reduction in 1-hexanol induced by early defoliation is correlated with the greater total soluble solids in must, where the defoliation treatment was performed, especially at pre-bloom. Conversely, Šuklje et al. [5] observed that the concentration of 1-hexanol was significantly increased by early defoliation although higher total soluble solids were measured in defoliation-treated wines than that in the control ones, which was in accordance with another study with grapes clarified as moderate maturity (°Brix of grape at 23–24, not included in this meta-analysis) [56]. Basal defoliation applied at the ripening stage also exhibits varied effects on 1-hexanol in wines from grapes with moderate maturity [2,45]. Other factors besides grape maturity, such as cultivar genotype, might mediate the behavior of basal defoliation in 1-hexanol in wine. The concentrations of (*E*)-3-hexenol and (*Z*)-3-hexenol have been found to range 3–191 and 10–94 μg/L in wine, respectively, and be much lower than their thresholds (400 and 1550 μg/L for (*E*)-3-hexenol and (*Z*)-3-hexenol, respectively) [57]. Thus, the flavor notes of the wines seem to be unaffected, although basal defoliation can exhibit varied effects on (*E*)-3-hexenol and (*Z*)-3-hexenol [28,29,30,56]. C_6_ aldehydes in grapes, such as hexanal and (*E*)-2-hexenal, have been found to be mostly transformed to their corresponding alcohols in wine during fermentation due to the activity of alcohol dehydrogenase (ADH) in yeast [58], and no C_6_ aldehydes have been reported in the studies included in this meta-analysis. Multiple C_6_ alcohols and aldehydes are precursors to hexyl acetate [59], which can be synthesized by alcohol acetyl-transferases (AATases) in yeast during fermentation [60]. Hexyl acetate contributes to “green apple” and sweet aromas of wine. Meta-analysis shows that defoliation has no significant effect on the concentrations of hexyl acetate in wine with low heterogeneity (Figure S3, SMD = −0.09, 95% CI = −0.56–0.38, *p* = 0.70, *p* for heterogeneity = 0.03, *I*^2^ = 43%). Several studies reported that different yeast strains affected the content of hexyl acetate in wine [61,62], so the yeast strain possibly contributes to the heterogeneity of meta-analysis for hexyl acetate.

#### 2.2.2. Higher Alcohols and Their Derived Acetates

Isobutanol, isoamyl alcohol, and *β*-phenylethanol are the most abundant higher alcohols in wine. Among them, isobutanol and isoamyl alcohol suppress fruity and woody notes but not leather/animal/ink notes and impart a negative aroma quality to wine [63]. The presence of *β*-phenylethanol can contribute to “honey and rose” aromas of wine. Higher alcohols in wine are produced from amino acid catabolic metabolism as well as sugar anabolic metabolism. Briefly, α-keto acids are generated through amino acid deamination (the Ehrlich pathway) [64] or biosynthesized from hexoses (the anabolic pathway) [65] and are then enzymatically decarboxylated to their corresponding aldehydes, which can be reduced to higher alcohols.

**Figure 5:**
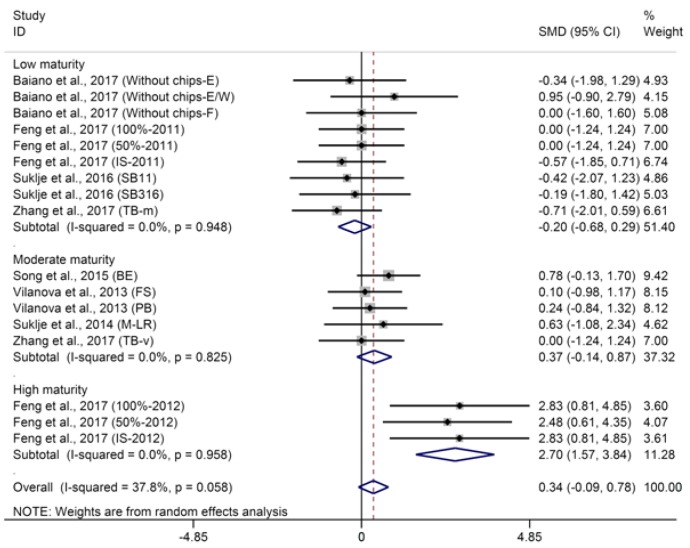
Meta-analysis for the effects of basal defoliation on *β*-phenylethanol in wine, of which trials were categorized based on the berry maturity level (low maturity of °Brix < 23, moderate maturity of 23 < °Brix < 25, high maturity of °Brix > 25).

Generally, production of higher alcohol in wine is negatively correlated with yeast assimilable nitrogen (YAN) in must [66]. Furthermore, there is a close relationship between some higher alcohols in wine and some specific amino acids in must; for instance, the levels of *β*-phenylethanol and methionol in wine are closely related to the levels of phenylalanine and methionine in must, respectively [67]. Martin et al. [68] measured similar YAN levels in exposed and shaded berries in two of three years. Moreover, although sunlight exposure can reduce the content of amino acids in berries as opposed to that in shaded berries [23,24], a greater proportion of higher alcohols are synthesized via the anabolic pathway of hexoses, versus the catabolism of amino acids via the Ehrlich pathway [65], so it appears that neither isobutanol or isoamyl alcohol in wine are affected by basal defoliation according to the result of meta-analysis (Figure S4, SMD of −0.03 and −0.18, 95% CI of −0.37–0.32 and −0.14–0.50, *p* of 0.87 and 0.27 for isobutanol and isoamyl alcohol, respectively), which found no significant heterogeneity (Figure S4, *p* for heterogeneity of 0.73 and 0.75 for isobutanol and isoamyl alcohol, respectively, *I*^2^ of 0% for both isobutanol and isoamyl alcohol). Kozina et al. [25] also reported no differences between control and defoliated wines for the concentrations of isobutanol and isoamyl alcohol. However, it is significant that two other studies not included in this meta-analysis found that early basal defoliation can significantly enhance the concentrations of isobutanol and isoamyl alcohol in wine [27,56]. Aggregated results of the meta-analysis shows that basal defoliation does not impose a significant effect on *β*-phenylethanol in wine (Figure 5, SMD = 0.34, 95% CI = −0.09–0.78, *p* = 0.12, *p* for heterogeneity = 0.06, *I*^2^ = 38%). Surprisingly, meta-regression analysis suggests that the berry maturity level is the main source of heterogeneity and explains 100% of the heterogeneous source of the pooled effect (*p* = 0.001). Further subgroup analysis of berry maturity shows that basal defoliation has no significant effect on the content of *β*-phenylethanol in wines from grapes with low and moderate maturity (Figure 5, SMD of −0.20 and 0.37, 95% CI of −0.68–0.29 and −0.14–0.87, *p* of 0.43 and 0.82 for *β*-phenylethanol in wines from grapes with low and moderate maturity, respectively) but significantly increases the content of *β*-phenylethanol in wines from grapes with high maturity (Figure 5, SMD = 2.70, 95% CI = 1.57–3.84, *p* < 0.001). These three berry maturity level subgroup meta-analyses for *β*-phenylethanol are not heterogeneous (*p* for heterogeneity of 0.95, 0.83, and 0.96 for low, moderate, and high maturity subgroup meta-analyses for *β*-phenylethanol, respectively, *I*^2^ = 0% for all three subgroup meta-analyses for *β*-phenylethanol). Therefore, the effect of basal defoliation on *β*-phenylethanol in wine can be mediated by grape maturity.

**Figure 6:**
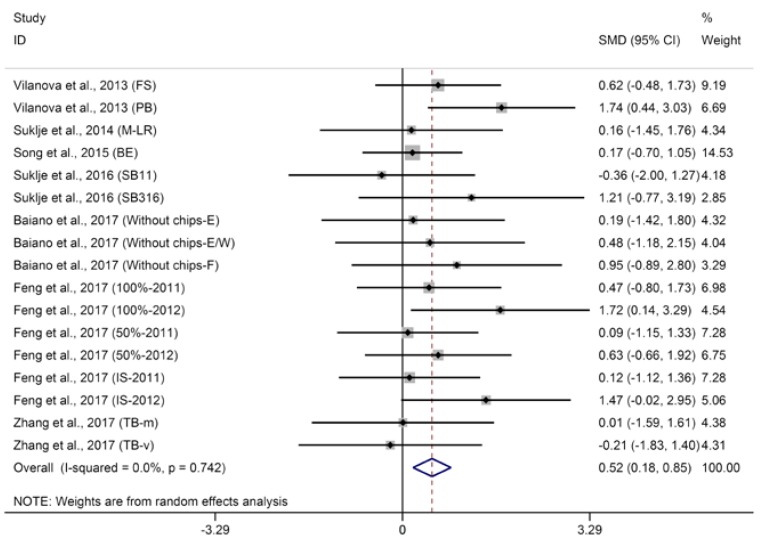
Meta-analysis for the effects of basal defoliation on isoamyl acetate in wine.

**Figure 7:**
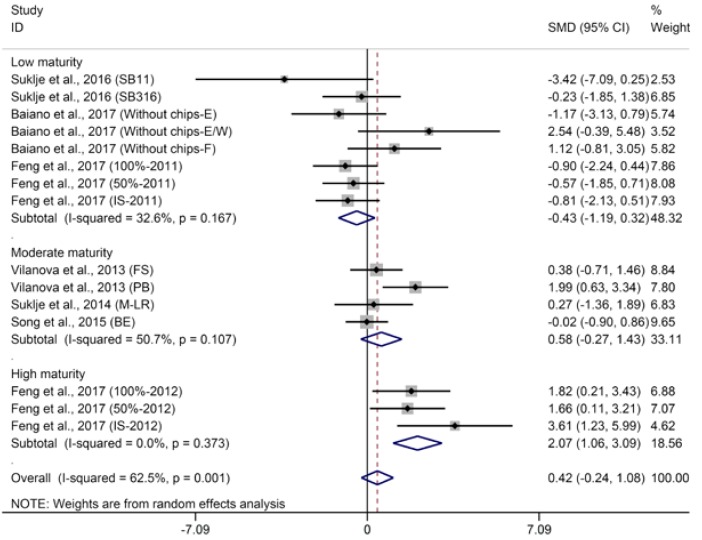
Meta-analysis for the effects of basal defoliation on 2-phenyl acetate in wine, of which trials were categorized based on the berry maturity level (low maturity of °Brix < 23, moderate maturity of 23 < °Brix < 25, high maturity of °Brix > 25).

Higher alcohol acetates (HAAs), including isobutyl acetate, isoamyl acetate, 2-phenylethyl acetate, contribute to the fruity aroma of wine. Meta-analysis suggests that basal defoliation decreases the concentrations of isobutyl acetate in wine but not significantly (Figure S5, SMD = 0.43, 95% CI = −0.07–0.94, *p* = 0.09, *p* for heterogeneity = 0.22, *I*^2^ = 24%), which agrees with the studies of Kozina et al. [25] and Hernandez-Orte et al. [26] not included in the meta-analysis. Regarding isoamyl acetate in wine, basal defoliation significantly increased its concentration (Figure 6, SMD = 0.52, 95% CI = 0.18–0.85, *p* = 0.002, *p* for heterogeneity = 0.74, *I*^2^ = 0%). Similarly, Verzera et al. [29] observed that basal defoliation increased the concentration of isoamyl acetate in wines from grapes harvested at first date (°Brix of grape approximately 24.8), and Morena et al. [56] observed that basal defoliation increased the concentration of isoamyl acetate in wine. The aggregated results of meta-analysis indicate that basal defoliation does not produce a significant effect on the concentration of 2-phenylethyl acetate in wine (Figure 7, SMD = 0.42, 95% CI = −0.22–1.05, *p* = 0.20, *p* for heterogeneity < 0.001, *I*^2^ = 63%). Meta-regression suggests that the berry maturity level is the major contributor to the heterogeneity of meta-analysis for 2-phenylethyl acetate in wine and explained 89.3% of heterogeneity (*p* = 0.004). Subgroup analysis based on berry maturity level indicates that basal defoliation has no significant effect on 2-phenylethyl acetate in wines from grapes with low and medium maturity (Figure 7, SMD of −0.43 and 0.58, 95% CI of −1.19–0.32 and −0.27–1.43, *p* of 0.26 and 0.18, *p* for heterogeneity of 0.17 and 0.11, *I*^2^ of 33% and 51% for 2-phenylethyl acetate in wines from grapes with low and moderate maturity, respectively), but significantly increases the content of 2-phenylethyl acetate in wines from grapes with high maturity (Figure 7, SMD = 2.07, 95% CI = 1.06–3.09, *p* < 0.001, *p* for heterogeneity = 0.37, *I*^2^ = 0%). Not surprisingly, since *β*-phenylethanol is the precursor of phenyl acetate, subgroup analysis of 2-phenyl acetate shows results similar to those of β-phenylethanol; the effects of defoliation on phenyl acetate appear to vary in wines from grapes with different maturities.

#### 2.2.3. Fatty Acids and Their Derived Ethyl Esters

Hexanoic acid, octanoic acid, and decanoic acid are common volatile fatty acids in wine, and octanoic acid is more abundant than the other two compounds. These volatile fatty acids contribute to fatty, rancid, and cheese aromas.

**Figure 8:**
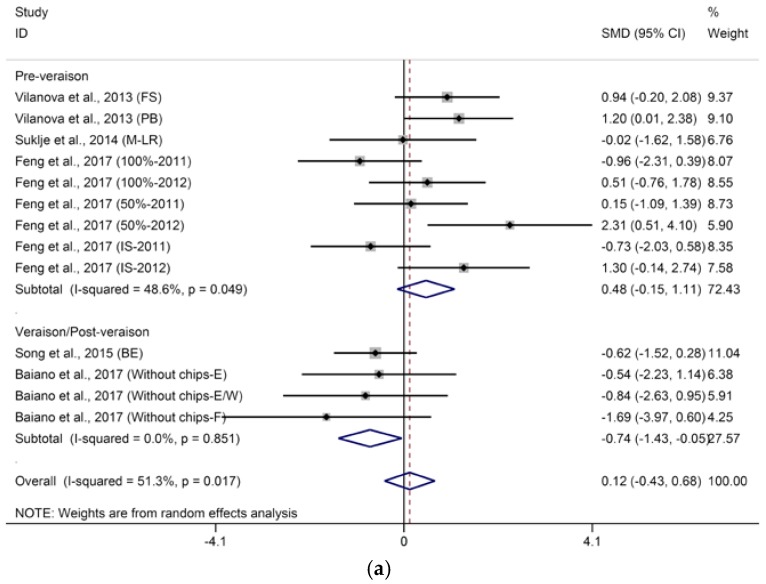

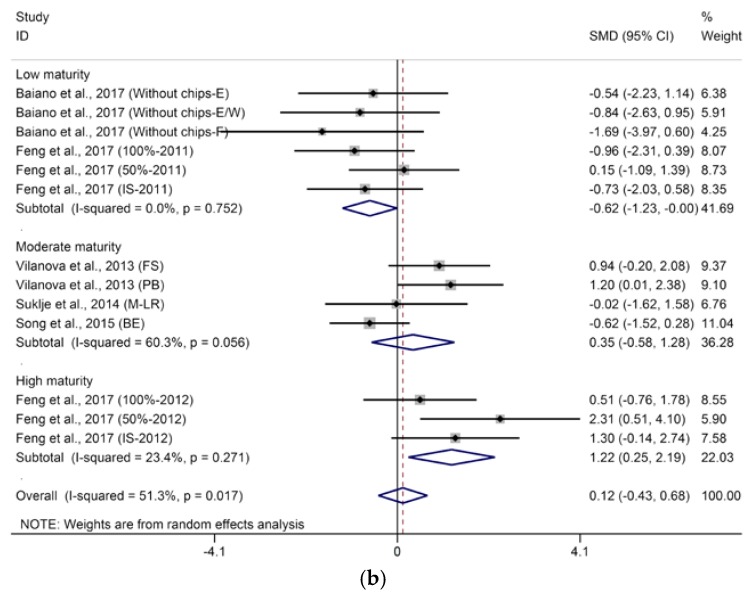
Meta-analysis for the effects of basal defoliation on decanoic acid in wine, of which trials were categorized based on the (**a**) timing of basal defoliation (pre-veraison and veraison/post-veraison) and (**b**) berry maturity level (low maturity of °Brix < 23, moderate maturity of 23 < °Brix < 25, high maturity of °Brix > 25).

Meta-analysis shows that defoliation has no significant effect on the concentration of hexanoic acid in wine (Figure S6a, SMD = 0.38, 95% CI = −0.01–1.94, *p* = 0.05, *p* for heterogeneity = 0.75 *I*^2^ = 0%). Contradictory to the current meta-analysis results, Verzera et al. [29] observed that defoliation applied at fruit set significantly increased the content of hexanoic acid in wines from grapes harvested at first date (°Brix of grape approximately 24.8), and Moreno et al. [56] documented that defoliation applied at pre-bloom significantly increased the content of hexanoic acid in wines in two of three seasons. Octanoic acid in wines was not significantly affected by basal defoliation (Figure S6b, SMD = −0.01, 94% CI = −0.47–0.44, *p* = 0.95, *p* for heterogeneity = 0.08, *I*^2^ = 38%). Additionally, sensitivity analysis shows that *I*^2^, for the test of heterogeneity, clearly decreased to 13% after the study of Song et al. is excluded [45], who observed that basal defoliation significantly decreased the concentration of octanoic acid*.* Comparatively, Song et al. [45], Verzera et al. [29], and Moreno et al. [56] each reported an opposite effect of basal defoliation on octanoic acid in wines. Regarding decanoic acid in wine, the overall effect of basal defoliation on its concentration was not significant with moderate heterogeneity (Figure 8, SMD = 0.12, 95% CI = −0.43–0.68, *p* = 0.66, *p* for heterogeneity = 0.02, *I*^2^ = 51%). Timing of basal defoliation and berry maturity level both contributed to the heterogeneity of meta-analysis for decanoic acid (*p* of 0.041 and 0.009, respectively). Subgroup analysis based on the timing of basal defoliation applied shows that early basal defoliation seems to have no remarkable effect on the content of decanoic acid in wine (Figure 8a, SMD = 0.48, 95% CI = −0.15–1.11, *p* = 0.13, *p* for heterogeneity = 0.05, *I*^2^ = 49%). Late basal defoliation reduces the contents of decanoic acid in wines (Figure 8a, SMD = −0.74, 95% CI = −1.43–0.05, *p* = 0.04, *p* for heterogeneity = 0.85, *I*^2^ = 0%). A further subgroup analysis based on berry maturity level was conducted to investigate the effects of basal defoliation on decanoic acid in wines from grapes with varying maturity levels. These results show that basal defoliation leads to a reduction in decanoic acid in wine from grapes with low maturity (Figure 7b, SMD = −0.62, 95% CI = −1.23–0.00, *p* = 0.05, *p* for heterogeneity = 0.75, *I*^2^ = 0%) but induces an increase in decanoic acid level in wines from grapes with high maturity (Figure 8b, SMD = 1.22, 95% = 0.25–2.19, *p* = 0.01, *p* for heterogeneity = 0.27, *I*^2^ = 23%). Verzera et al. [29] also observed inconsistent effects on the contents of decanoic acid imposed by early defoliation in wines from grapes harvested at different times, and they found that early defoliation significantly increased the content of decanoic acid in wine from grapes harvested at first date (°Brix of grape approximately 24.8) but had no significant effect on wines from grapes harvested at second date (°Brix of grape approximately 24.6). Moreover, volatile fatty acids have been found to be generally produced as minor byproducts of fatty metabolism in yeast during fermentation, which is sensitive to nutrient status, oxygen availability, and temperature [69]; therefore, all these factors might mediate the effects of basal defoliation on volatile fatty acids levels in wine.

**Figure 9:**
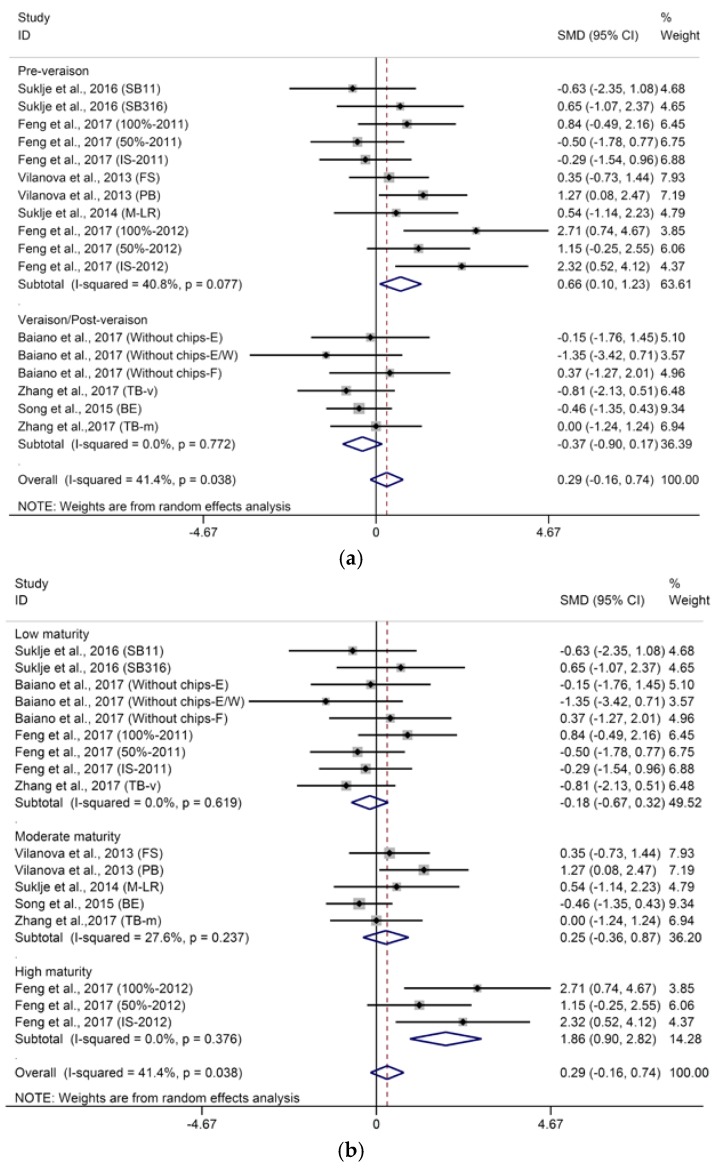
Meta-analysis for the effects of basal defoliation on ethyl octanoate in wine, of which trials were categorized based on the (**a**) timing of basal defoliation (pre-veraison and veraison/post-veraison), and (**b**) berry maturity level (low maturity of °Brix < 23, moderate maturity of 23 < °Brix < 25, high maturity of °Brix > 25).

Ethyl esters of fatty acids (EEFAs) are generally catalyzed by acyl-CoA/ethanol O-acyltransferase enzymes (Eeb1p and Eht1p) via condensation of fatty acid-CoA with ethanol during fermentation [21]. EEFAs are mainly responsible for the fruity aromas of wine. Ethyl butyrate, ethyl isobutyrate, ethyl hexanoate, ethyl isovalerate, ethyl octanoate, and ethyl decanoate are generally considered important odorants due to their relatively high typical concentration and odor activity values in wine. Meta-analysis suggests that defoliation significantly improves the concentration of ethyl butyrate in wine (SMD = 0.59, 95% CI = 0.12–1.06, *p* = 0.01, *p* for heterogeneity = 0.04, *I*^2^ = 41%); however, sensitivity analysis, after the observations from the 100% defoliation treatment in the study of Feng et al. [28] are excluded, shows that no significant effect of defoliation on the concentration of ethyl butyrate has been observed (Figure S7a, SMD = 0.36, 95% CI = −0.03–0.76, *p* = 0.07, *p* for heterogeneity = 0.26, *I*^2^ = 17%). Although basal defoliation tends to induce slight increases for the concentrations of ethyl isobutyrate, ethyl hexanoate, ethyl isovalerate, and ethyl decanoate in wine, these increases are not significant (Figure S7b–e). Regarding ethyl octanoate, the aggregated results show that basal defoliation does not significantly affect its concentration in wine with low heterogeneity (Figure 9, SMD = 0.29, 95% CI = −0.16–0.74, *p* = 0.21, *p* for heterogeneity = 0.04, *I*^2^ = 41%). Meta-regression analysis shows that the timing of basal defoliation applied and berry maturity level are the main contributors to the heterogeneity (*p* of 0.028 and 0.007, respectively). Subgroup analysis based on the timing of basal defoliation applied show that late defoliation has no significant effect on the concentration of ethyl octanoate in wine (Figure 9a, SMD = −0.41, 95% CI = −0.95–0.12, *p* = 0.18, *p* for heterogeneity = 0.77, *I*^2^ = 0%). Furthermore, subgroup analysis based on berry maturity level indicates that the concentrations of ethyl octanoate in wines from grapes with low and moderate maturity are not affected by basal defoliation, but are significantly increased in wines from grapes with high maturity (Figure 9b). In addition to the studies included in meta-analysis, Verzera et al. [29] observed that fruit set defoliation significantly increased the concentration of ethyl octanoate in wine from the first harvest date (°Brix of grape approximately 24.8), whereas it did not exhibit a significant effect on ethyl octanoate in wines from the second harvest date (°Brix of grape approximately 24.6). Moreno et al. [56] observed that pre-bloom defoliation induced an increase in most ethyl esters in wine. Based on the results above, the authors can infer that the timing of basal defoliation applied and berry maturity are both responsible for the concentration of ethyl octanoate in wine, but how these two factors influence the effect of basal defoliation on the ethyl octanoate level in wine needs to be further investigated.

## 3. Materials and Methods

### 3.1. Data Selection

Papers were collected using the search terms “wine” AND (“leaf removal” OR “defoliation”) AND (“aroma*” OR “volatile*”)” on Google Scholar, and the records were included until December 2017. Studies were withheld only if they met following criteria:

- There were ≥2 repetitions.
- Control and treatment measurements were reported.
- Means, standard errors, or *p* values were reported. Standard error could be estimated from *p*-values [70]. Additionally, 1% of the mean was used as an estimate of standard error to calculate the effect size for a few compounds with no variance reported in some studies [71].
- Basal defoliation was applied rather than other techniques to improve cluster exposure such as shoot thinning.

### 3.2. Database

Based on the selection criteria, nine studies were selected for this meta-analysis [2,5,27,28,30,31,38,45,53]. Data reported in these nine studies were obtained from tables or extracted from plots using ImageJ (Version 1.51K; National Institute of Health, Bethesda, MD, USA). Volatile compounds were classified into two groups: (1) varietal flavors and aromas, including C_13_-norisoprenoids, terpenes, and methoxypyrazines; and (2) flavors and aromas formed during fermentation, including C_6_ compounds, higher alcohols, and their acetates, fatty acids, and their ethyl esters. The study characteristics (authors, publication year, and replicates), grape cultivar, difference of berry maturity between control and defoliation treatment (MDf), berry maturity level (BM), timing (DT), and severity (DS) of basal defoliation for each study were recorded (Table S1). Data regarding all different grape varieties or clones, timings of basal defoliation, levels of basal defoliation, and vintages in one study were extracted and separated into different independent trials.

### 3.3. Data Analysis

Meta-analysis and statistical analyses were conducted using Stata program (Version 12.0; Stata Corporation, College Station, TX, USA) and REVMAN software (Version 5.0; Cochrane Collaboration, Oxford, UK). The standard mean differences (SMDs) and 95% confidence intervals (CIs) were estimated from each trial for the effects of basal defoliation on volatile compounds in wines by using random effect models. Heterogeneity of basal defoliation effects across trials was tested with Hedge’s test (*p* < 0.05), which examines the null hypothesis that all studies are evaluating the same effect. The *I*^2^ statistics, a quantitative measure of inconsistency across trials, were also examined, as they provide the proportion of total variation in study estimates due to heterogeneity. The authors considered that trials with an *I*^2^ value of 25–50% as having low heterogeneity, trials with an *I*^2^ of 50–75% as having moderate heterogeneity and trials with an *I*^2^ > 75% as having high heterogeneity. Meta-regression was conducted to explore potential explanations for heterogeneity within the significance of berry maturity differences among control and defoliation treatments, berry maturity level, timing of basal defoliation applied, and the level of basal defoliation applied as covariate, and further subgroup analysis was conducted based on these covariates. Additionally, potential sources of heterogeneity were also identified by sensitivity analysis to investigate the influence of specific trials on the overall pooled estimate. To facilitate the meta-regression analysis, differences in berry maturity among control and defoliation treatments, berry maturity level, as well as timing and severity of basal defoliation were set as covariates, and were quantitatively defined as the following criteria:

- MDf: 1 = “total soluble solids showed no significant difference between control and treated berries”; 2 = “total soluble solids in treated berries were significantly higher than that in control berries”.
- BM: 1 (low maturity) = “°Brix < 23”; 2 (moderate maturity) = “23 < °Brix < 25”; 3 (high maturity) = “°Brix > 25”.
- DT: 1 = “pre-veraison”; 2 = “veraison/post-veraison”.
- DS: 1 = “all leaves removed from the base of shoot to the node just above the apical cluster”; 2 = “not all leaves removed from the base of shoot to the node just above the apical cluster”.

Moreover, due to the limited information of climate in the published studies and no explicit criteria for grape cultivar, the authors narratively discussed the influence of basal defoliation on wine aromas in different cultivars and climates.

## 4. Conclusions

The current meta-analysis quantitatively reviews the effects of basal defoliation on the volatile compounds in wine by the synthesis of previous published studies and revealed potential factors relative to discrepancies across these studies. Summarily, timing of basal defoliation plays an important role in the concentration of varietal aromas in wine. Pre-veraison defoliation can induce an increase in *β*-damascenone and linalool as well as a reduction in IBMP. The effects of basal defoliation on certain volatile compounds relative to fermentation aromas in wine (1-hexanol, *β*-phenylethanol, 2-phenylethyl acetate, decanoic acid, and ethyl octanoate) depend on grape maturity. Additionally, it was significant that isobutanol, isoamyl alcohol, hexanoic acid, and octanoic acid as well as ethyl isobutyrate, ethyl hexanoate, ethyl isovalerate, and ethyl decanoate in wine were not affected markedly by basal defoliation. The current meta-analysis results serve as a reminder that great attention should be given to the timing of basal defoliation and grape maturity in the application of basal defoliation. Certainly, there are other factors such as cultivar and climate conditions that are responsible for the behavior of basal defoliation on wine aromas, though these factors were not quantitatively assessed in this meta-analysis. Due to the current meta-analysis results being based on the limited available studies, understanding the precise effect of basal defoliation on wine aromas remains a great challenge for viticulturists and winemakers. More rigorous and carefully designed experiments should be conducted in the future.

A future area with significant need is to assess the effect of basal defoliation on wine sensory. Odor activity values (OAVs) of volatile compounds in wine and sensory analysis could help researchers to understand whether changes in the concentrations of volatile compounds in wine caused by basal defoliation can be perceived. Furthermore, economic justification should be considered also when growers apply basal defoliation. Regarding the control of bunch diseases, basal defoliation, especially if applied at an early stage, could be more effective than fungicide applications. Previous studies have demonstrated that basal defoliation could save 27% on costs [16]. Additionally, another study reported that pre-bloom defoliation had no negative influence on yield and effectively reduced the cost to produce one unit of total skin anthocyanins in a warm region [72]. Basal defoliation appeared to benefit cultivators in these cases. However, Geller et al. [73] reported that inclusion of mechanical basal defoliation added an additional cost of $65/hectare, while having no beneficial effects on yield components or berry composition. Wine bouquet is of great importance to wine quality; however, to the best of the author’s knowledge, no study has assessed whether the gains in wine sensory quality can offset the cost of basal defoliation. Someday, studies on wine sensory quality should also take the cost of basal defoliation into account.

## Supplementary Materials

The following are available online at http://www.mdpi.com/1420-3049/23/4/779/s1. Figure S1: Meta-analysis for the effect of basal defoliation on *β*-ionone in wine; Figure S2: Meta-analysis for the effect of basal defoliation on geraniol in wine; Figure S3: Meta-analysis for the effect of basal defoliation on hexyl acetate in wine; Figure S4: Meta-analysis for the effect of basal defoliation on (a) isobutanol and (b) isoamyl alcohol in wine; Figure S5: Meta-analysis for the effect of basal defoliation on isobutyl acetate in wine; Figure S6: Meta-analysis for the effect of basal defoliation on (a) hexanoic acid and (b) octanoic acid in wine; Figure S7: Meta-analysis for the effect of basal defoliation on (a) ethyl butyrate, (b) ethyl isobutyrate, (c) ethyl hexanoate, (d) ethyl isovalerate and (e) ethyl decanoate in wine; Table S1: Characteristics of the 9 studies included in the meta-analysis.

## References

[1] Alessandrini M., Battista F., Panighel A., Flamini R., Tomasi D. (2018). Effect of pre-bloom leaf removal on grape aroma composition and wine sensory profile of Semillon cultivar. J. Sci. Food Agric..

[2] Zhang P., Wu X., Needs S., Liu D., Fuentes S., Howell K. (2017). The influence of apical and basal defoliation on the canopy structure and biochemical composition of *Vitis vinifera* cv. Shiraz grapes and wine. Front. Chem..

[3] Mosetti D., Herrera J.C., Sabbatini P., Green A., Alberti G., Peterlunger E., Lisjak K., Castellarin S.D. (2016). Impact of leaf removal after berry set on fruit composition and bunch rot in ‘Sauvignon blanc’. Vitis.

[4] Young P.R., Eyeghe-Bickong H.A., du Plessis K., Alexandersson E., Jacobson D.A., Coetzee Z., Deloire A., Vivier M.A. (2016). Grapevine plasticity in response to an altered microclimate: Sauvignon Blanc modulates specific metabolites in response to increased berry exposure. Plant Physiol..

[5] Šuklje K., Antalick G., Coetzee Z., Schmidtke L.M., Baša Česnik H., Brandt J., du Toit W.J., Lisjak K., Deloire A. (2014). Effect of leaf removal and ultraviolet radiation on the composition and sensory perception of *Vitis vinifera* L. cv. Sauvignon Blanc wine. Aust. J. Grape Wine Res..

[6] Kuhn N., Guan L., Dai Z.W., Wu B.-H., Lauvergeat V., Gomès E., Li S.-H., Godoy F., Arce-Johnson P., Delrot S. (2014). Berry ripening: Recently heard through the grapevine. J. Exp. Bot..

[7] Poni S., Casalini L., Bernizzoni F., Civardi S., Intrieri C. (2006). Effects of early defoliation on shoot photosynthesis, yield components, and grape composition. Am. J. Enol. Vitic..

[8] Diago M.P., Ayestaran B., Guadalupe Z., Poni S., Tardaguila J. (2012). Impact of prebloom and fruit set basal leaf removal on the flavonol and anthocyanin composition of Tempranillo grapes. Am. J. Enol. Vitic..

[9] Frinoi T., Zhuang S., Palliotti A., Sivilotti P., Falchi R., Sabbatini P. (2017). Leaf removal and cluster thinning efficiencies are highly modulated by environmental conditions in cool climate viticulture. Am. J. Enol. Vitic..

[10] Keller M. (2010). Managing grapevine to optimize fruit development in a challenging environment: A climate change primer for viticulturists. Aust. J. Grape Wine Res..

[11] Palliotti A., Panara F., Sivestroni O., Lanari V., Sabbatini P., Howell G.S., Gatti M., Poni S. (2013). Influence of mechanical postveraison leaf removal apical to the cluster zone on delay of fruit ripening in Sangiovese (*Vitis vinifera* L.) grapevines. Aust. J. Grape Wine Res..

[12] Poni S., Gatti M., Bernizzoni F., Civardi S., Bobeica N., Magnanini E., Palliotti A. (2013). Late leaf removal aimed at delaying ripening in cv. Sangiovese: Physiological assessment and vine performance. Aust. J. Grape Wine Res..

[13] Filippetti I., Movahed N., Allegro G., Valentini G., Pastore C., Colucci E., Intrieri C. (2015). Effect of post-veraison source limitation on the accumulation of sugar, anthocyanins and seed tannins in *Vitis vinifera* cv. Sangiovese berries. Aust. J. Grape Wine Res..

[14] Bergqvist J., Dokoozlian N., Ebisuda N. (2001). Sunlight exposure and temperature effects on berry growth and composition of Cabernet Sauvignon and Grenache in the central San Joaquin Valley of California. Am. J. Enol. Vitic..

[15] Chorti E., Guidoni S., Ferrandino A., Novello V. (2010). Effect of different cluster sunlight exposure levels on ripening and anthocyanin accumulation in Nebbiolo grapes. Am. J. Enol. Vitic..

[16] Sternad Lemut M., Sivilotti P., Butinar L., Laganis J., Vrhovsek U. (2015). Pre-flowering leaf removal alters grape microbial population and offers good potential for a more sustainable and cost-effective management of a Pinot Noir vineyard. Aust. J. Grape Wine Res..

[17] Kotseridis Y., Georgiadou A., Tikos P., Kallithraka S., Koundouras S. (2012). Effects of severity of post-flowering leaf removal on berry growth and composition of three red *Vitis vinifera* L. cultivars grown under semiarid conditions. J. Agric. Food Chem..

[18] Feng H., Yuan F., Skinkis P.A., Qian M.C. (2015). Influence of cluster zone leaf removal on Pinot noir grape chemical and volatile composition. Food Chem..

[19] Sun R.-Z., Cheng G., Li Q., He Y.-N., Wang Y., Lan Y.-B., Li S.-Y., Zhu Y.-R., Song W.-F., Zhang X. (2017). Light-induced variation in phenolic compounds in Cabernet Sauvignon grapes (*Vitis vinifera* L.) involves extensive transcriptome reprogramming of biosynthetic enzymes, transcription factors, and phytohormonal regulators. Front. Plant Sci..

[20] Rapp A., Mandery H. (1986). Wine aroma. Cell. Mol. Life Sci..

[21] Styger G., Prior B., Bauer F.F. (2011). Wine flavor and aroma. J. Ind. Microbiol. Biotechnol..

[22] Xu X.-Q., Cheng G., Duan L.-L., Jiang R., Pan Q.-H., Duan C.-Q., Wang J. (2015). Effect of training systems on fatty acids and their derived volatiles in Cabernet Sauvignon grapes and wines of the north foot of Mt. Tianshan. Food Chem..

[23] Gregan S.M., Wargent J.J., Liu L., Shinkle J., Hofmann R., Winefield C., Trought M., Jordan B. (2012). Effects of solar ultraviolet radiation and canopy manipulation on the biochemical composition of Sauvignon Blanc grapes. Aust. J. Grape Wine Res..

[24] Friedel M., Stoll M., Patz C., Will F., Dietrich H. (2015). Impact of light exposure on fruit composition of white ‘Riesling’ grape berries (*Vitis vinifera* L.). Vitis.

[25] Kozina B., Karoglan M., Herjavec S., Jeromel A., Orlić S. (2008). Influence of basal leaf removal on the chemical composition of Sauvignon Blanc and Riesling wines. J. Food Agric. Environ..

[26] Hernandez-Orte P., Concejero B., Astrain J., Lacau B., Cacho J., Ferreira V. (2015). Influence of viticulture practices on grape aroma precursors and their relation with wine aroma. J. Sci. Food Agric..

[27] Šuklje K., Antalick G., Buica A., Langlois J., Coetzee Z.A., Gouot J., Schmidtke L.M., Deloire A. (2016). Clonal differences and impact of defoliation on Sauvignon blanc (*Vitis vinifera* L.) wines: A chemical and sensory investigation. J. Sci. Food Agric..

[28] Feng H., Skinkis P.A., Qian M.C. (2017). Pinot noir wine volatile and anthocyanin composition under different levels of vine fruit zone leaf removal. Food Chem..

[29] Verzera A., Tripodi G., Dima G., Condurso C., Scacco A., Cincotta F., Giglio D.M.L., Santangelo T., Sparacio A. (2016). Leaf removal and wine composition of *Vitis vinifera* L. cv. Nero d’Avola: The volatile aroma constituents. J. Sci. Food Agric..

[30] Vilanova M., Diago M.P., Genisheva Z., Oliveira J.M., Tardaguila J. (2012). Early leaf removal impact on volatile composition of Tempranillo wines. J. Sci. Food Agric..

[31] Baiano A., Mentana A., Quinto M., Centonze D., Previtali M.A., Varva G., Del Nobile M.A., De Palma L. (2017). Volatile composition and sensory profile of wines obtained from partially defoliated vines: The case of Nero di Troia wine. Eur. Food Res. Technol..

[32] Winterhalter P., Sefton M., Williams P. (1990). Volatile C_13_-norisoprenoid compounds in Riesling wine are generated from multiple precursors. Am. J. Enol. Vitic..

[33] Mathieu S., Terrier N., Procureur J., Bigey F., Günata Z. (2005). A carotenoid cleavage dioxygenase from *Vitis vinifera* L.: Functional characterization and expression during grape berry development in relation to C_13_-norisoprenoid accumulation. J. Exp. Bot..

[34] Lashbrooke J.G., Young P.R., Dockrall S.J., Vasanth K., Vivier M.A. (2013). Functional characterisation of three members of the *Vitis vinifera* L. carotenoid cleavage dioxygenase gene family. BMC Plant Biol..

[35] Pineau B., Barbe J.-C., Van Leeuwen C., Dubourdieu D. (2007). Which impact for *β*-damascenone on red wines aroma?. J. Agric. Food Chem..

[36] Gómez-Míguez M.J., Cacho J.F., Ferreira V., Vicario I.M., Heredia F.J. (2007). Volatile components of Zalema white wines. Food Chem..

[37] Kotseridis Y., Baumes R.L., Bertrand A., Skouroumounis G.K. (1999). Quantitative determination of *β*-ionone in red wines and grapes of Bordeaux using a stable isotope dilution assay. J. Chromatogr. A.

[38] Kwasniewski M.T., Vanden Heuvel J.E., Pan B.S., Sacks G.L. (2010). Timing of cluster light environment manipulation during grape development affects C_13_ norisoprenoid and carotenoid concentrations in Riesling. J. Agric. Food Chem..

[39] Hirschberg J. (2001). Carotenoid biosynthesis in flowering plants. Curr. Opin. Plant Biol..

[40] Marais J., Van Wyk C., Rapp A. (1992). Effect of sunlihgt and shade on norisoprenoid levels in maturing Weisser Riesling and Chenin Blanc grapes and Weisser Riesling wines. S. Afr. J. Enol. Vitic..

[41] Mateo J.J., Jiménez M. (2000). Monoterpenes in grape juice and wines. J. Chromatogr. A.

[42] Marais J. (1983). Terpenes in the aroma of grapes and wines: A review. S. Afr. J. Enol. Vitic..

[43] Schreier P., Jennings W.G. (1979). Flavor composition of wines: A review. Crit. Rev. Food Sci. Nutr..

[44] Sasaki K., Takase H., Matsuyama S., Kobayashi H., Matsuo H., Ikoma G., Takata R. (2016). Effect of light exposure on linalool biosynthesis and accumulation in grape berries. Biosci. Biotechnol. Biochem..

[45] Song J., Smart R., Wang H., Dambergs B., Sparrow A., Qian M.C. (2015). Effect of grape bunch sunlight exposure and UV radiation on phenolics and volatile composition of *Vitis vinifera* L. cv. Pinot noir wine. Food Chem..

[46] Fang Y., Qian M.C. (2006). Quantification of selected aroma-active compounds in Pinot Noir wines from different grape maturities. J. Agric. Food Chem..

[47] Maicas S., Mateo J.J. (2005). Hydrolysis of terpenyl glycosides in grape juice and other fruit juices: A review. Appl. Microbiol. Biotechnol..

[48] Koch A., Ebeler S.E., Williams L.E., Matthews M.A. (2012). Fruit ripening in *Vitis vinifera*: Light intensity before and not during ripening determines the concentration of 2-methoxy-3-isobutylpyrazine in Cabernet Sauvignon berries. Physiol. Plant..

[49] Allen M.S., Lacey M.J., Harris R.L.N., Brown W.V. (1991). Contribution of methoxypyrazines to Sauvignon Blanc wine aroma. Am. J. Enol. Vitic..

[50] Hein K., Ebeler S.E., Heymann H. (2009). Perception of fruity and vegetative aromas in red wine. J. Sens. Stud..

[51] Ryona I., Pan B.S., Sacks G.L. (2009). Rapid measurement of 3-alkyl-2-methoxypyrazine content of winegrapes to predict levels in resultant wines. J. Agric. Food Chem..

[52] Scheiner J.J., Sacks G.L., Pan B., Ennahli S., Tarlton L., Wise A., Lerch S.D., Heuvel J.E.V. (2010). Impact of severity and timing of basal leaf removal on 3-isobutyl-2-methoxypyrazine concentrations in red winegrapes. Am. J. Enol. Vitic..

[53] Sivilotti P., Herrera J.C., Lisjak K., Baša Česnik H., Sabbatini P., Peterlunger E., Castellarin S.D. (2016). Impact of leaf removal, applied before and after flowering, on anthocyanin, tannin, and methoxypyrazine concentrations in ‘Merlot’ (*Vitis vinifera* L.) grapes and wines. J. Agric. Food Chem..

[54] Ameye M., Allmann S., Verwaeren J., Smagghe G., Haesaert G., Schuurink R.C., Audenaert K. (2017). Green leaf volatile production by plants: A meta-analysis. New Phytol..

[55] Herraiz T., Herraiz M., Reglero G., Martin-Alvarez P.J., Cabezudo M.D. (1990). Changes in the composition of alcohols and aldehydes of C_6_ chain length during the alcoholic fermentation of grape must. J. Agric. Food Chem..

[56] Moreno D., Valdés E., Uriarte D., Gamero E., Talaverano I., Vilanova M. (2017). Early leaf removal applied in warm climatic conditions: Impact on Tempranillo wine volatiles. Food Res. Int..

[57] Guth H. (1997). Quantitation and sensory studies of character impact odorants of different white wine varieties. J. Agric. Food Chem..

[58] De Smidt O., du Preez J.C., Albertyn J. (2008). The alcohol dehydrogenases of *Saccharomyces cerevisiae*: A comprehensive review. FEMS Yeast Res..

[59] Dennis E.G., Keyzers R.A., Kalua C.M., Maffei S.M., Nicholson E.L., Boss P.K. (2012). Grape contribution to wine aroma: Production of hexyl acetate, octyl acetate, and benzyl acetate during yeast fermentation is dependent upon precursors in the must. J. Agric. Food Chem..

[60] Lilly M., Bauer F.F., Lambrechts M.G., Swiegers J.H., Cozzolino D., Pretorius I.S. (2006). The effect of increased yeast alcohol acetyltransferase and esterase activity on the flavour profiles of wine and distillates. Yeast.

[61] Estevez P., Gil M.L., Falque E. (2004). Effects of seven yeast strains on the volatile composition of Palomino wines. Int. J. Food Sci. Technol..

[62] Torrens J., Urpi P., Riu-Aurnatell M., Vichi S., Lopez-Tamames E., Buxaderas S. (2008). Different commercial yeast strains affecting the volatile and sensory profile of cava base wine. Int. J. Food Microbiol..

[63] De-la-Fuente-Blanco A., Sáenz-Navajas M.-P., Ferreira V. (2016). On the effects of higher alcohols on red wine aroma. Food Chem..

[64] Hazelwood L.A., Daran J.M., van Maris A.J.A., Pronk J.T., Dickinson J.R. (2008). The Ehrlich pathway for fusel alcohol production: A century of research on *Saccharomyces cerevisiae* metabolism. Appl. Environ. Microbiol..

[65] Nisbet M.A., Tobias H.J., Brenna J.T., Sacks G.L., Mansfield A.K. (2014). Quantifying the contribution of grape hexoses to wine volatiles by high-precision [U^13^C]-glucose tracer studies. J. Agric. Food Chem..

[66] Bell S.J., Henschke P.A. (2005). Implications of nitrogen nutrition for grapes, fermentation and wine. Aust. J. Grape Wine Res..

[67] Hernández-Orte P., Cacho J.F., Ferreira V. (2002). Relationship between varietal amino acid profile of grapes and wine aromatic composition. Experiments with model solutions and chemometric study. J. Agric. Food Chem..

[68] Martin D., Grose C., Fedrizzi B., Stuart L., Albright A., McLachlan A. (2016). Grape cluster microclimate influences the aroma composition of Sauvignon blanc wine. Food Chem..

[69] Waterhouse A.L., Sacks G.L., Jeffery D.W. (2016). Understanding Wine Chemistry.

[70] Higgins J.P., Green S. (2011). Cochrane Handbook for Systematic Reviews of Interventions.

[71] Rowen E., Kaplan I. (2016). Eco-evolutionary factors drive induced plant volatiles: A meta-analysis. New Phytol..

[72] Cook M.G., Zhang Y., Nelson C.J., Gambetta G., Kennedy J.A., Kurtural S.K. (2015). Anthocyanin composition of Merlot is Ameliorated by light microclimate and irrigation in central California. Am. J. Enol. Vitic..

[73] Geller J.P., Kurtural S.K. (2013). Mechanical canopy and crop-load management of Pinot Gris in a warm climate. Am. J. Enol. Vitic..

