# Supplementary material for: Effects of Basal Defoliation on Wine Aromas: A Meta-Analysis
Source: Molecules. 2018 Mar 28;23(4):779. doi: 10.3390/molecules23040779 (PMC6017958; doi:10.3390/molecules23040779)
Supplement: Supplementary file 1 [file molecules-23-00779-s001.pdf]

# Effects of Basal Defoliation on Wine Aromas: A Meta-Analysis

**Supplementary Materials:**

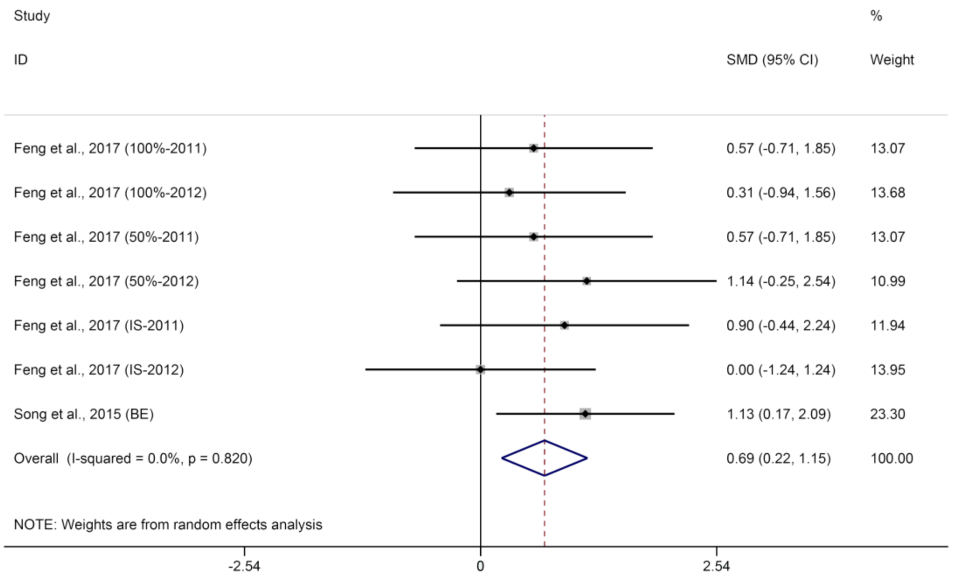


**Figure S1.** Meta-analysis for the effects of basal defoliation on *β*-ionone in wine.

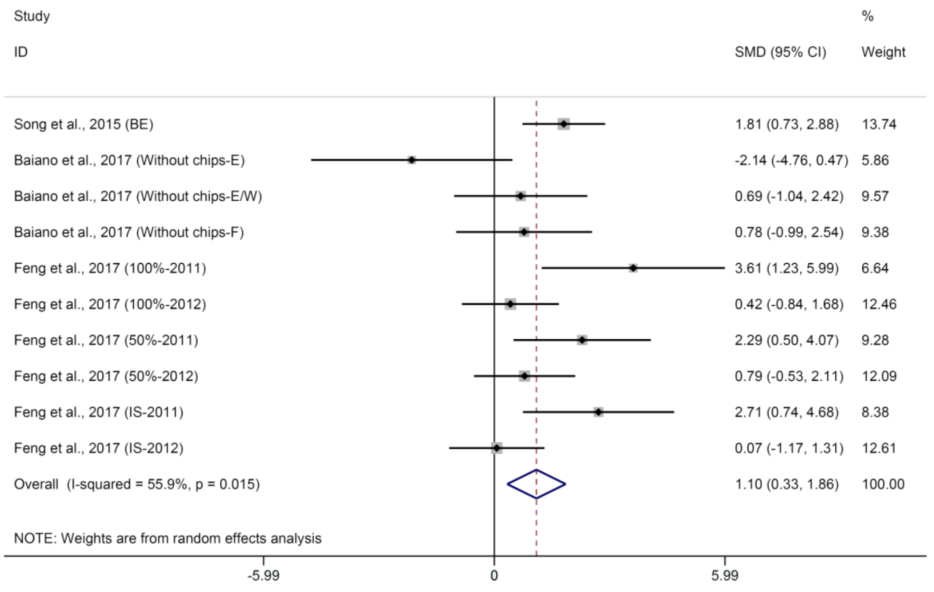


**Figure S2.** Meta-analysis for the effects of basal defoliation on geraniol in wine.

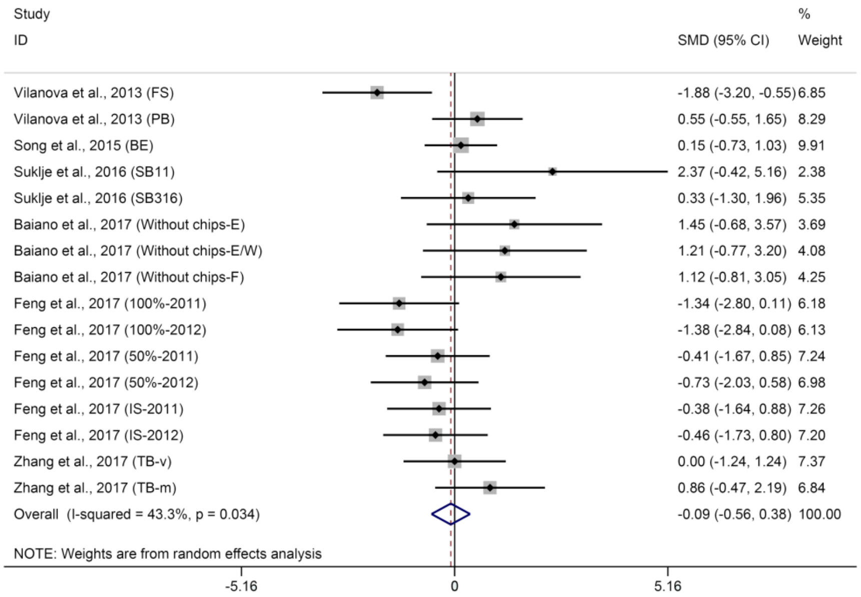


**Figure S3.** Meta-analysis for the effects of basal defoliation on hexyl acetate in wine.

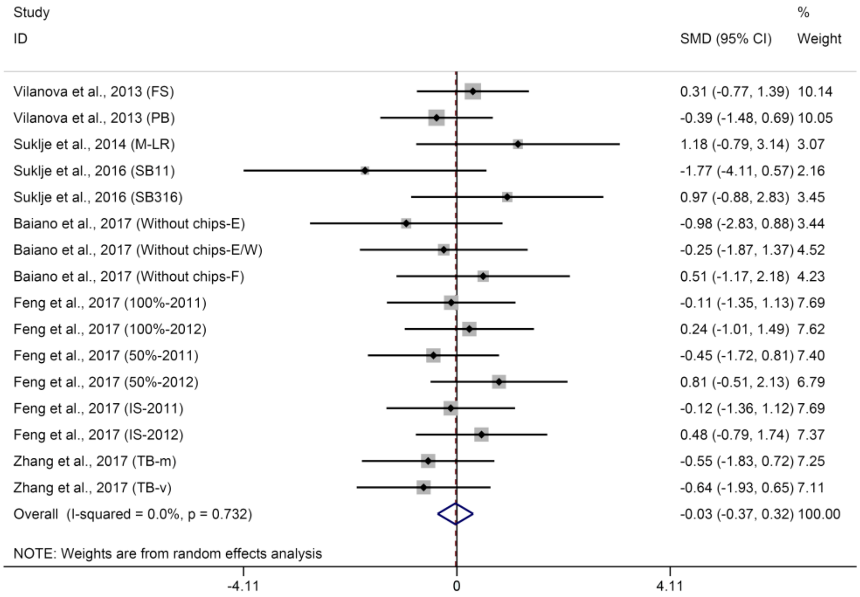


(**a**)

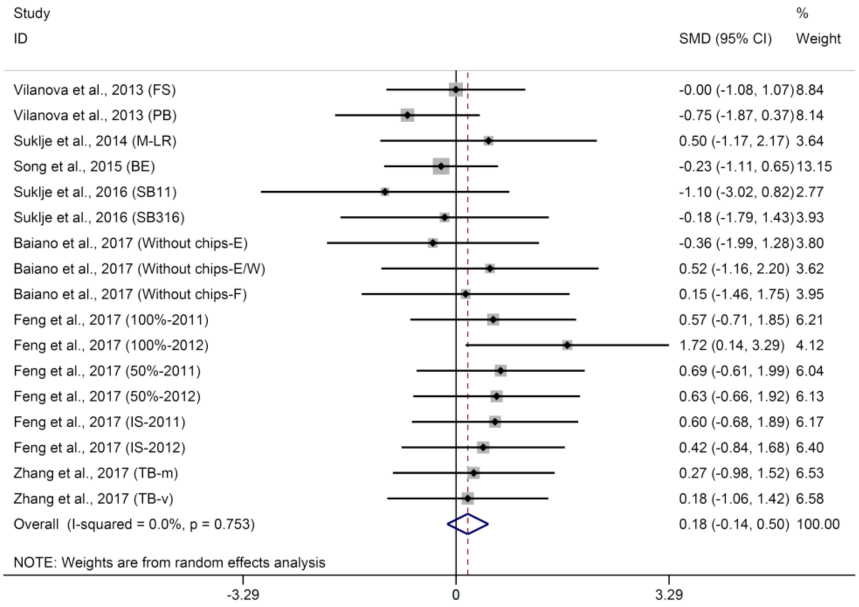


(**b**)

**Figure S4.** Meta-analysis for the effects of basal defoliation on (a) isobutanol and (b) isoamyl alcohol in wine.

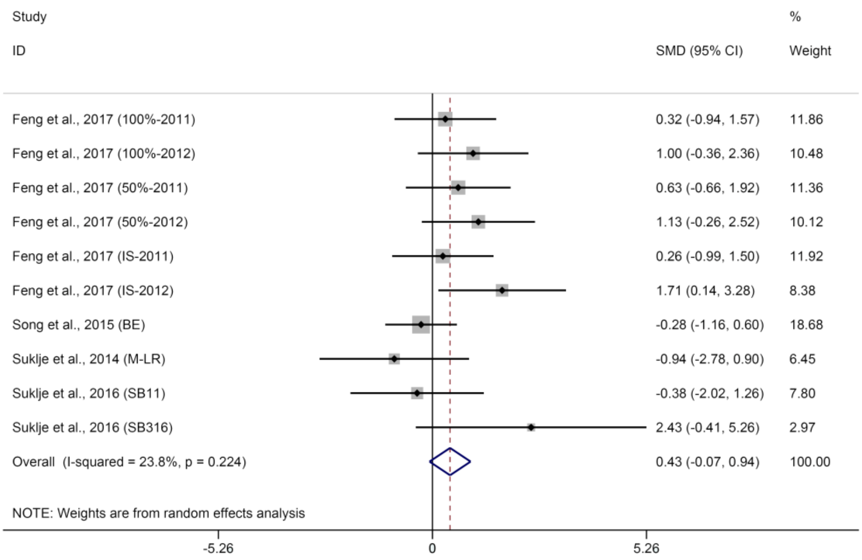


**Figure S5.** Meta-analysis for the effects of basal defoliation on isobutyl acetate in wine.

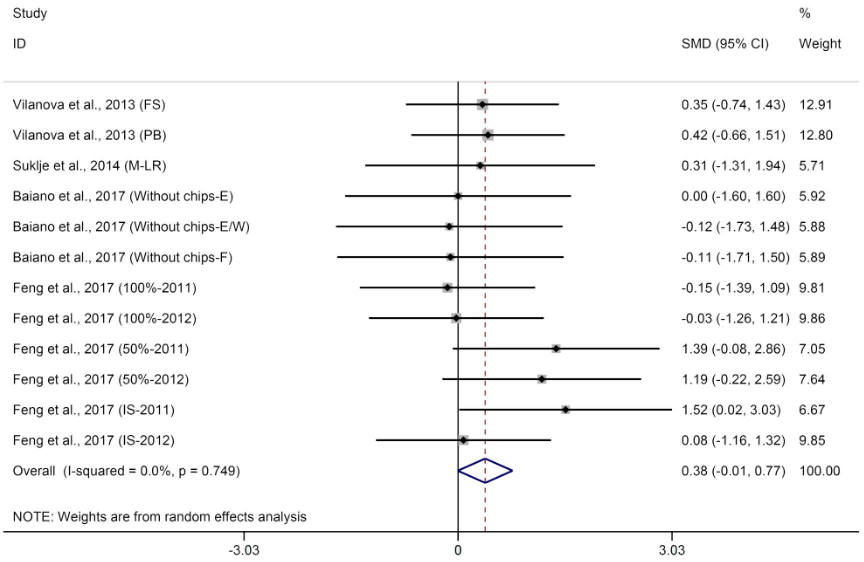


(**a**)

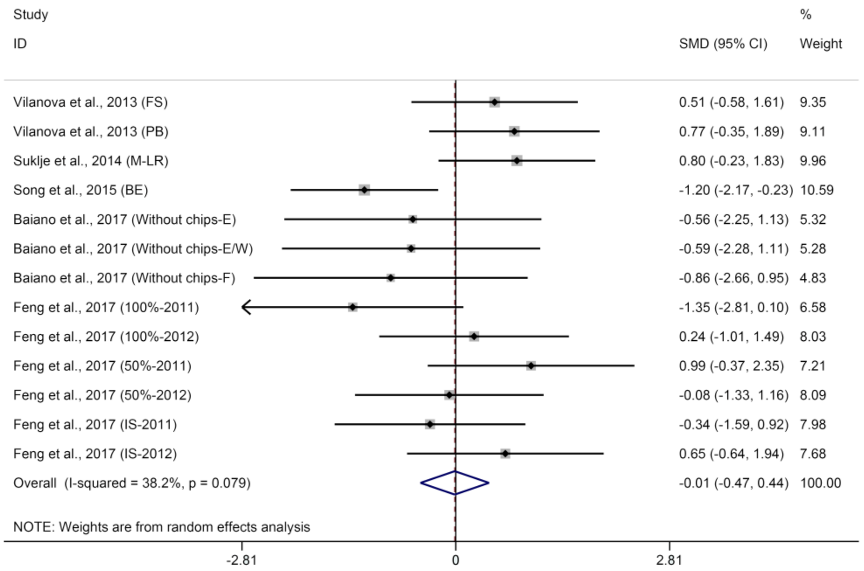


(**b**)

**Figure S6.** Meta-analysis for the effects of basal defoliation on (a) hexanoic acid and (b) octanoic acid in wine.

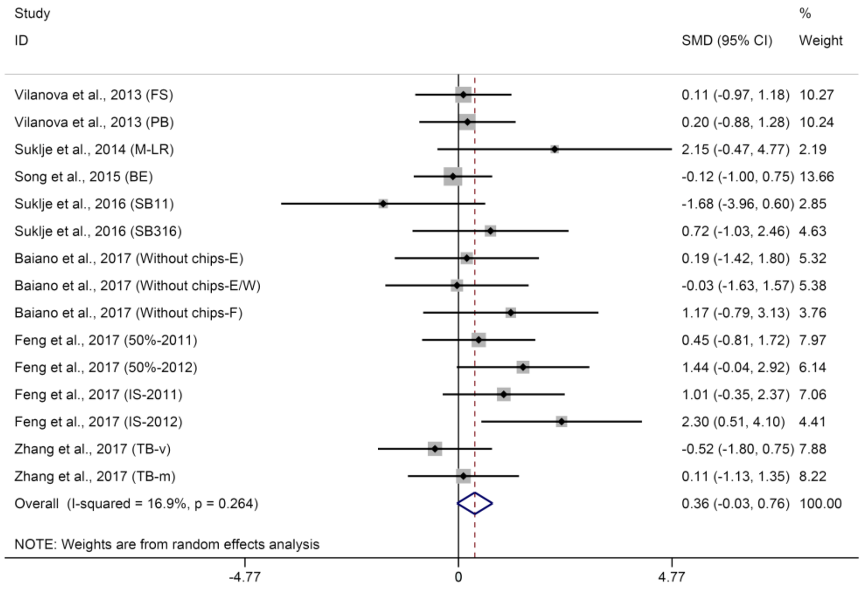
Study
ID
SMD (95% CI)
%
Weight
Vilanova et al., 2013 (FS) 0.11 (-0.97, 1.18) 10.27
Vilanova et al., 2013 (PB) 0.20 (-0.88, 1.28) 10.24
Suklje et al., 2014 (M-LR) 2.15 (-0.47, 4.77) 2.19
Song et al., 2015 (BE) -0.12 (-1.00, 0.75) 13.66
Suklje et al., 2016 (SB11) -1.68 (-3.96, 0.60) 2.85
Suklje et al., 2016 (SB316) 0.72 (-1.03, 2.46) 4.63
Baiano et al., 2017 (Without chips-E) 0.19 (-1.42, 1.80) 5.32
Baiano et al., 2017 (Without chips-E/W) -0.03 (-1.63, 1.57) 5.38
Baiano et al., 2017 (Without chips-F) 1.17 (-0.79, 3.13) 3.76
Feng et al., 2017 (50%-2011) 0.45 (-0.81, 1.72) 7.97
Feng et al., 2017 (50%-2012) 1.44 (-0.04, 2.92) 6.14
Feng et al., 2017 (IS-2011) 1.01 (-0.35, 2.37) 7.06
Feng et al., 2017 (IS-2012) 2.30 (0.51, 4.10) 4.41
Zhang et al., 2017 (TB-v) -0.52 (-1.80, 0.75) 7.88
Zhang et al., 2017 (TB-m) 0.11 (-1.13, 1.35) 8.22
Overall (I-squared = 16.9%, p = 0.264) 0.36 (-0.03, 0.76) 100.00
NOTE: Weights are from random effects analysis
-4.77
0
4.77

(**a**)

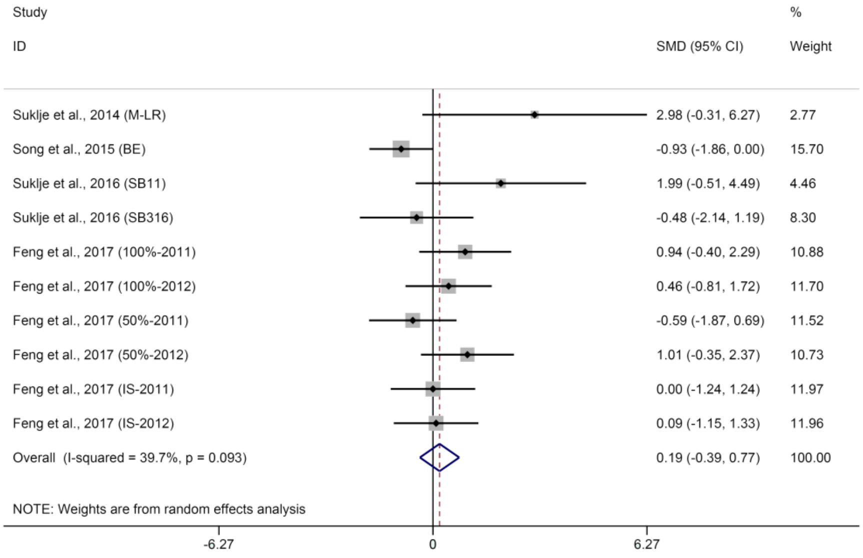
Study
ID
SMD (95% CI)
%
Weight
Suklje et al., 2014 (M-LR) 2.98 (-0.31, 6.27) 2.77
Song et al., 2015 (BE) -0.93 (-1.86, 0.00) 15.70
Suklje et al., 2016 (SB11) 1.99 (-0.51, 4.49) 4.46
Suklje et al., 2016 (SB316) -0.48 (-2.14, 1.19) 8.30
Feng et al., 2017 (100%-2011) 0.94 (-0.40, 2.29) 10.88
Feng et al., 2017 (100%-2012) 0.46 (-0.81, 1.72) 11.70
Feng et al., 2017 (50%-2011) -0.59 (-1.87, 0.69) 11.52
Feng et al., 2017 (50%-2012) 1.01 (-0.35, 2.37) 10.73
Feng et al., 2017 (IS-2011) 0.00 (-1.24, 1.24) 11.97
Feng et al., 2017 (IS-2012) 0.09 (-1.15, 1.33) 11.96
Overall (I-squared = 39.7%, p = 0.093) 0.19 (-0.39, 0.77) 100.00
NOTE: Weights are from random effects analysis
-6.27
0
6.27

(**b**)

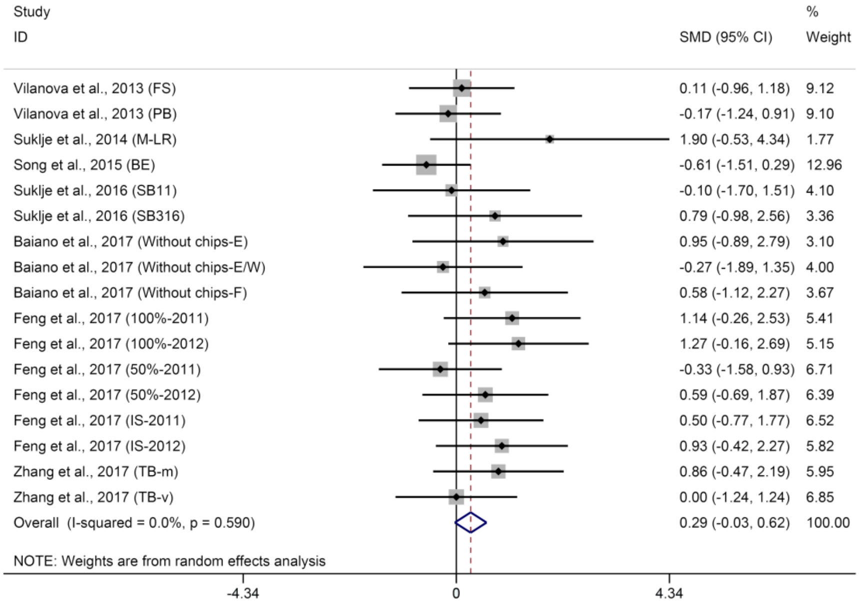

Study
ID
SMD (95% CI)
%
Weight
Vilanova et al., 2013 (FS) 0.11 (-0.96, 1.18) 9.12
Vilanova et al., 2013 (PB) -0.17 (-1.24, 0.91) 9.10
Suklje et al., 2014 (M-LR) 1.90 (-0.53, 4.34) 1.77
Song et al., 2015 (BE) -0.61 (-1.51, 0.29) 12.96
Suklje et al., 2016 (SB11) -0.10 (-1.70, 1.51) 4.10
Suklje et al., 2016 (SB316) 0.79 (-0.98, 2.56) 3.36
Baiano et al., 2017 (Without chips-E) 0.95 (-0.89, 2.79) 3.10
Baiano et al., 2017 (Without chips-E/W) -0.27 (-1.89, 1.35) 4.00
Baiano et al., 2017 (Without chips-F) 0.58 (-1.12, 2.27) 3.67
Feng et al., 2017 (100%-2011) 1.14 (-0.26, 2.53) 5.41
Feng et al., 2017 (100%-2012) 1.27 (-0.16, 2.69) 5.15
Feng et al., 2017 (50%-2011) -0.33 (-1.58, 0.93) 6.71
Feng et al., 2017 (50%-2012) 0.59 (-0.69, 1.87) 6.39
Feng et al., 2017 (IS-2011) 0.50 (-0.77, 1.77) 6.52
Feng et al., 2017 (IS-2012) 0.93 (-0.42, 2.27) 5.82
Zhang et al., 2017 (TB-m) 0.86 (-0.47, 2.19) 5.95
Zhang et al., 2017 (TB-v) 0.00 (-1.24, 1.24) 6.85
Overall (I-squared = 0.0%, p = 0.590) 0.29 (-0.03, 0.62) 100.00
NOTE: Weights are from random effects analysis
-4.34
0
4.34


(c)

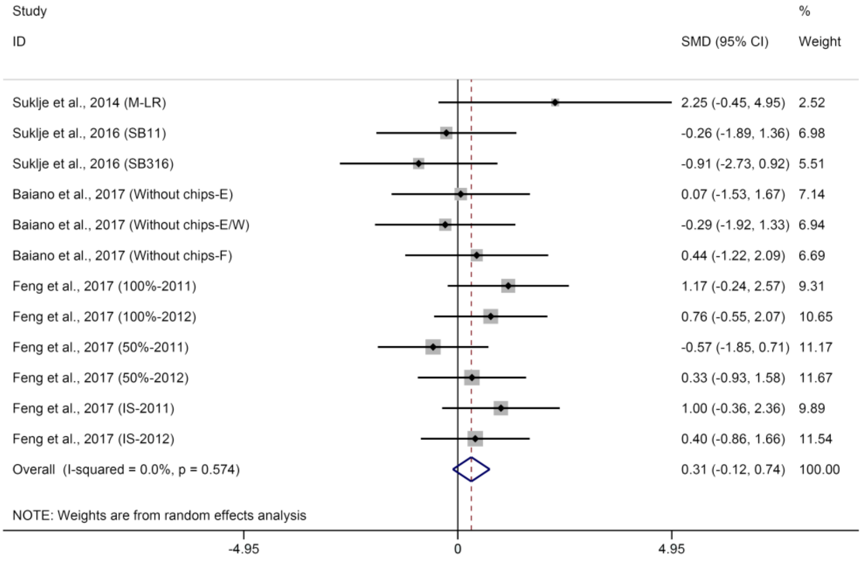

Study
ID
SMD (95% CI)
%
Weight
Suklje et al., 2014 (M-LR) 2.25 (-0.45, 4.95) 2.52
Suklje et al., 2016 (SB11) -0.26 (-1.89, 1.36) 6.98
Suklje et al., 2016 (SB316) -0.91 (-2.73, 0.92) 5.51
Baiano et al., 2017 (Without chips-E) 0.07 (-1.53, 1.67) 7.14
Baiano et al., 2017 (Without chips-E/W) -0.29 (-1.92, 1.33) 6.94
Baiano et al., 2017 (Without chips-F) 0.44 (-1.22, 2.09) 6.69
Feng et al., 2017 (100%-2011) 1.17 (-0.24, 2.57) 9.31
Feng et al., 2017 (100%-2012) 0.76 (-0.55, 2.07) 10.65
Feng et al., 2017 (50%-2011) -0.57 (-1.85, 0.71) 11.17
Feng et al., 2017 (50%-2012) 0.33 (-0.93, 1.58) 11.67
Feng et al., 2017 (IS-2011) 1.00 (-0.36, 2.36) 9.89
Feng et al., 2017 (IS-2012) 0.40 (-0.86, 1.66) 11.54
Overall (I-squared = 0.0%, p = 0.574) 0.31 (-0.12, 0.74) 100.00
NOTE: Weights are from random effects analysis
-4.95
0
4.95


(d)

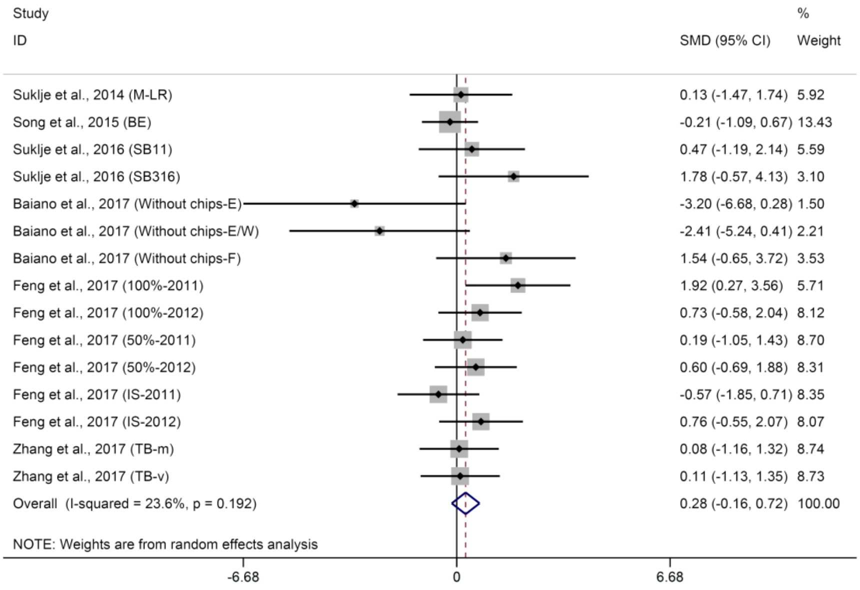


(**e**)

**Figure S7.** Meta-analysis for the effects of basal defoliation on (a) ethyl butyrate, (b) ethyl isobutyrate, (c) ethyl hexanoate, (d) ethyl isovalerate and (e) ethyl decanoate in wine.

**Table S1.** Characteristics of the nine studies included in the meta-analysis.

| Author, publication year | Treatment ID | Replicates | Grape variety | MDf[1] | BM[2] | DT[3] | DS[4] |
|---|---|---|---|---|---|---|---|
| Kwasniewski et al., 2010 | 2days PBS | 2 | Riesling | ns | low | pre-veraison | 75% |
| | 33days PBS | 2 | Riesling | ns | low | pre-veraison | 75% |
| | 68days PBS | 2 | Riesling | ns | low | pre-veraison | 75% |
| Vilanova et al., 2012 | PB | 10 | Tempranillo | higher | moderate | pre-veraison | 100% |
| | FS | 10 | Tempranillo | higher | moderate | pre-veraison | 100% |
| Suklje et al., 2014 | M-LR | 3 | Sauvignon Blanc | higher | moderate | pre-veraison | 100% |
| Song et al., 2015 | BS | 10 | Pinot Noir | higher | moderate | veraison | 100% |
| Sivilotti., 2016 | BF | 3 | Merlot | ns | low | pre-veraison | 75% |
| | AF | 3 | Merlot | ns | low | pre-veraison | 75% |
| Suklje et al., 2016 | SB11 | 3 | Sauvignon Blanc | higher | low | pre-veraison | 100% |
| | SB316 | 3 | Sauvignon Blanc | ns | low | pre-veraison | 100% |
| Baiano et al., 2017 | Without chips-E | 3 | Nero di Troia | ns | low | veraison | 75% |
| | Without chips-E/W | 3 | Nero di Troia | ns | low | veraison | 75% |
| | Without chips-F | 3 | Nero di Troia | higher | low | veraison | 100% |
| Feng et al., 2017 | 100%-2011 | 5 | Pinot Noir | ns | low | pre-veraison | 100% |
| | 50%-2011 | 5 | Pinot Noir | ns | low | pre-veraison | 50% |
| | IS-2011 | 5 | Pinot Noir | ns | low | pre-veraison | 100% |

| | | | | | | | |
|---|---|---|---|---|---|---|---|
| | 100%-2012 | 5 | Pinot Noir | ns | high | pre-veraison | 100% |
| | 50%-2012 | 5 | Pinot Noir | ns | high | pre-veraison | 50% |
| | IS-2013 | 5 | Pinot Noir | ns | high | pre-veraison | 100% |
| Zhang et al., 2017 | TB-v | 5 | Shiraz | ns | moderate | veraison | 75% |
| | TB-m | 5 | Shiraz | ns | low | post-veraison | 75% |

[1] difference of berry maturity between control and defoliation treatment; [2] berry maturity level; [3] timing of basal defoliation; [4] severity of basal defoliation.
